# Physician Adherence to Treat-to-Target and Practice Guidelines in Rheumatoid Arthritis

**DOI:** 10.3390/jcm8091416

**Published:** 2019-09-08

**Authors:** Bogdan Batko, Krzysztof Batko, Marcin Krzanowski, Zbigniew Żuber

**Affiliations:** 1 Faculty of Medicine and Health Sciences, Andrzej Frycz Modrzewski University, Gustawa Herlinga-Grudźińskiego 1 St, 30-705 Cracow, Poland; 2 Department of Rheumatology, J. Dietl Specialist Hospital, Skarbowa 1 St, 31-121 Cracow, Poland; 3 Chair and Head of Nephrology, Jagiellonian University Medical College, Kopernika St 15c, 31-501 Cracow, Poland; 4 Department of Pediatrics, Faculty of Medicine and Health Sciences, Andrzej Frycz Modrzewski University, Gustawa Herlinga-Grudźińskiego 1 St, 30-705 Cracow, Poland; 5 Ward for Older Children with Neurology and Rheumatology Subdivision, St. Louis Regional Specialised Children’s Hospital, 31-503 Cracow, Poland

**Keywords:** rheumatologists, arthritis, rheumatoid, physicians, practice patterns, physicians’, guideline adherence, treat-to-target

## Abstract

Principles of treat-to-target (T2T) have been widely adopted in both multinational and regional guidelines for rheumatoid arthritis (RA). Several questionnaire studies among physicians and real-world data have suggested that an evidence–practice gap exists in RA management. Investigating physician adherence to T2T, which requires a process measure, is difficult. Different practice patterns among physicians are observed, while adherence to protocolized treatment declines over time. Rheumatologist awareness, agreement, and claims of adherence to T2T guidelines are not always consistent with medical records. Comorbidities, a difficult disease course, communication barriers, and individual preferences may hinder an intensive, proactive treatment stance. Interpreting deviations from protocolized treatment/T2T guidelines requires sufficient clinical context, though higher adherence seems to improve clinical outcomes. Nonmedical constraints in routine care may consist of barriers in healthcare structure and socioeconomic factors. Therefore, strategies to improve the institution of T2T should be tailored to local healthcare. Educational interventions to improve T2T adherence among physicians may show a moderate, although beneficial effect. Meanwhile, a proportion of patients with inadequately controlled RA exists, while management decisions may not be in accordance with T2T. Physicians tend to be aware of current guidelines, but their institution in routine practice seems challenging, which warrants attention and further study.

## 1. Treatment of Rheumatoid Arthritis to Target—What Are the Benefits?

Treat-to-target (T2T) refers to a set of principles that guide physician decision making by aiming to achieve prompt and effective control of the inflammatory process in rheumatoid arthritis (RA) [1], which has led to its endorsement in international recommendations [2]. The T2T strategy includes: Defining a target of therapy (most often remission); close and frequent assessment of disease activity with validated composite measures; regular adjustment of treatment plan if said target is not reached; consideration of patients’ clinical characteristics; and finally, their involvement in treatment decisions and planning [2]. The rationale can be drawn from evidence supporting intensive steering and medication over conventional strategies, which is substantiated by the benefits in clinical outcomes, including better physical function and reduced structural changes [3,4,5]. Systematic reviews have previously reported on the benefits of aiming for remission, which also includes a favorable effect on other elements of T2T, i.e., comorbidity, cardiovascular risk, and productivity [6]. Recent studies have shown that T2T yields higher remission rates and improves quality of life over routine care [7]. Other reports also indicate that utilizing a T2T approach holds high potential also in patients with inadequate response to conventional synthetic disease modifying anti-rheumatic drugs (DMARDs), which remain fundamentals in the current treatment armamentarium [8,9]. These studies underscore the importance of applying T2T in a personalized approach of care, for which there may be signs of suboptimal application.

In this report, we provide a narrative review of current literature examining the multiplanar aspects of T2T with perspectives on physician adherence and optimal management of RA.

## 2. Management of RA May Be Suboptimal

Observational data have previously suggested a substantial “evidence to practice gap”, i.e., in the institution of T2T, with the prevalence of inadequate disease control reported in a significant proportion of RA patients [10,11,12]. A recent systematic report on real-world remission rates under treat-to-target strategy shows an improvement over recent years, although sustained remission is still regarded as rare [13]. Taylor et al. provided an analysis of multinational data on RA management, reporting that no T2T approach was present in approximately half of patients, and only close to one third had remission as a chosen target [14]. When examining the subset of patients with RA diagnosis stated within two years, more patients lacked a treatment target, despite 38% of them suffering from moderate to severe RA. Meanwhile, the considerable majority of physicians in this subgroup (70%) were satisfied with RA control. Another recent study reported that among inadequate TNF inhibitor responders, suboptimal treatment decisions, such as lack of intensification, may be widely prevalent despite moderate to severe RA activity [15]. Although causal attribution cannot be strictly determined across these studies, it can be observed that the major goal of T2T, which is achieving remission, has not been reached for many patients. T2T has been adapted in numerous guidelines [12], which would imply that its application and core principles are widely adhered to, or at least aimed for. Meanwhile, observational studies have indicated that guideline adherence (including T2T elements) may be suboptimal in real-life, though it varies among particular guideline components [16]. While the choice of DMARDs in routine care is largely consistent with international recommendations [17,18], other studies indicate that nearly half of newly diagnosed RA patients are not initiated with a DMARD and receive symptomatic treatment [19]. This suggests that the choice of therapeutic agents is often concordant with recommendations, as compared to patterns of practice, which are more difficult to change. Understanding the practice patterns of rheumatologists may provide insights into unsatisfactory statistics of treatment in the real world, which is often discordant with efficacy reported in trials, and may relate to insufficient institution of T2T. 

## 3. Improving Physician Adherence to Treat-to-Target May Aid in Achieving Remission

Many factors affect the achievement of remission in RA, with high importance of early treatment with conventional synthetic DMARDs, or in some instances biologic DMARDs, are well established [9]. However, management of RA should also follow the T2T strategy; with an ongoing process of decisions aiming for remission shaping the treatment plan. Importantly, outside of protocolized trails these decisions are routinely at the treating rheumatologist’s discretion, though in theory dictated by practice guidelines. Considering studies among rheumatologists have shown that awareness and/or agreement with guidelines is not synonymous with their actual practice [18,20,21], investigating physician adherence is of particular interest. Kuusalo et al. performed a retrospective analysis of the new Finnish RA Combination Therapy (NEO-RACo) trial and reported that good physician adherence to remission-driven protocol was related to lower RA activity and even remission. When examining short and long-term predictors of remission, physician adherence was the most significant factor at three months, and the only determinant at a two year follow-up [22]. Notably, when considering these findings and that only four patients had a null (absolute) score for adherence, achieving optimal outcomes may require only a high rather than absolute degree of adherence, which is in line with other studies that have attempted to determine an adherence cut-off (respectively, 80% and 70% for remission and low disease activity using 28 joint count disease activity score (DAS28)) [23]. When examining the above and below optimal cut-off point for provider compliance and clinical outcomes, the odds of attaining DAS28 remission were near eight-fold greater. Although this illustrates the potential benefits of improving physician adherence, a cause-effect relationship cannot be easily determined.

## 4. Barriers to Guideline Implementation and Treat-to-Target

In the clinic, the treating physician often has to deal with limited time, communication difficulties, and healthcare regulations, which may all pose barriers to T2T implementation. In a discrete choice experiment for a model patient with RA, drug efficacy, economic aspects, and patient preferences were demonstrated to influence decisions among rheumatologists [24]. Studies have also indicated that both patient and physician preferences, as well as provider hesitation to therapy intensification are obstacles to T2T implementation [25]. These studies highlight the role of the payer and individual preference in the routine setting, which may also be constrained by a rheumatologist’s hesitation over therapy side-effects. Patient-related factors, comorbidities, and toxicities have all been previously associated with persistent or recurrence of T2T protocol deviation [26]. However, it should be noted that T2T strategy emphasizes the importance of patient outcomes and comorbidity. A personalized approach, which takes into account the patients clinical characteristics and wellbeing, while targeting remission, is in line with T2T. 

Electronic unavailability and time shortages have previously justified infrequent use of quantitative measures in routine care [27]. Assaying acute phase reactants also seems to be a substitute for composite indices [21], while remission is variably defined, frequently without validated scores [18]. Meanwhile, close and frequent monitoring of disease activity with established indices remains a staple of T2T. Other studies report that despite agreement with T2T, physicians may still not discuss treatment decisions with patients from their lack of understanding or inability to share decisions [28]. These findings show that certain suboptimal practice patterns may persist, despite an awareness of T2T. 

Vermeer et al. analyzed a random RA patient sample from the Dutch Rheumatoid Arthritis Monitoring registry (DREAM) cohort, which included several hospitals adopting a T2T strategy (for details, see Table 1) [29]. Adherence to treatment advice was reported as lower when remission was not present, though the justification was often thought to be valid. On a side note, analyses of Behandel Strategieen (BeSt) and IMPROVED revealed that targeting remission may lead to lower rates of adherence to DAS-steered protocol [30]. These studies may suggest that rheumatologists are less inclined to follow treatment guidelines when RA is not well-controlled, or the measures of disease activity do not reflect the physician’s view. Indeed, variability between personal judgment and composite indices, e.g., providers view of remission and presence of DAS28 ≤ 2.6, may contribute to findings of nonadherence [29]. 

In some countries, studies alarmingly indicate that treatment choices can be unjustified, patients are insufficiently monitored, and there is a lack of concordance with the current recommendations for practice [31]. Prior reports of the cross-country inequities in DMARD access provide insights into different local economic regulations [32]. These differences imply that rheumatologists may not always have access to the full treatment armamentarium, which limits the treatment adjustments for patients with active, unresponsive disease. Multinational surveys have indicated a wide variability in referral and early RA assessment pathways, while the impact and practice of guidelines may also be particularly country specific [33]. It seems that any interventions to improve T2T adherence will have to be revised by national expert societies, and tailored to the structure of local healthcare, which may also entail identifying different areas of “unmet needs” and assessing their priority.

## 5. Feasibility of Treat-to-Target and Decline in Adherence over Time

Versteeg et al. showed that a protocolized T2T strategy was successfully applied in the DREAM cohort, with favorable clinical outcomes reported in long term follow-up [34]. Importantly, contraindications and comorbidities did not lead to exclusion, with deviations from protocol allowed. This indicates that in order to follow T2T strategy, adjustments of treatment plan may be necessary to account for comorbidity and other patient-related factors. In a report from the Danish Registry for Biologic Therapies in Rheumatology (DANBIO), which was designed around data from clinical trials and incorporates automatic disease activity score (DAS) calculation, systematic monitoring was concluded to be feasible for routine care [35]. 

Although studies with a pre-defined protocol may not adequately reflect implementation of T2T/guidelines in routine practice, they lend credible evidence on the relationship between measures of T2T adherence and the providers’ behavior. Wabe et al. retrospectively studied an early RA population, where protocol deviation occurred in one fourth of visits (of which 43% justified clinically), was lowest during the first six months and considerably increased in subsequent timepoints of a three-year follow-up [26]. Patient-related factors, comorbidities, and toxicities were all significantly associated with persistent or recurrence of protocol deviation. Other studies have indicated that patient and physician preferences, as well as provider hesitation to therapy intensification are challenges to T2T implementation [25]. It has also been shown that the rate of treatment accelerations per visit decreased over time in both usual care and dedicated T2T centers [25]. Markusse et al. performed a post hoc analysis of the multicenter BeSt trial data and observed that the adherence rates declined substantially over a 10-year follow-up, down to approximately 60% [36]. Disagreement with subsequent treatment steps, reported by physicians in a questionnaire, was associated with a higher chance of protocol nonadherence. However, when considering the latter responses overall, only every fourth physician actually violated protocol. This highlights the difference between a standardized, controlled clinical trial and routine care scenarios, where the individual opinion is likely to take priority. 

## 6. Evaluating Physician Adherence is Difficult

Defining T2T adherence is difficult with it being a multifaceted strategy and process. Studies tend to focus on an aspect of guidelines, which may obscure the view on the general strategy. Escalas et al. reported prospective data from an early RA cohort where following analysis and use of a propensity model, adherence to three 2007 European League Against Rheumatism (EULAR) recommendations was determined. Although substantial for individual recommendations (>50%), the treatment adherence rate to all components was only 23% [37]. Other studies reported guideline adherence variability between 21 and 72%, with an approach to treat highest scoring indicators as a relative norm [16]. Not many studies have broadly examined physician adherence to several components of T2T at once, however, Yu et al. performed an analysis of medical records from the TRACTION trial and observed that physician adherence was sub-optimal when considering a score of T2T implementation, observing a wide variability in T2T component across visits (details, Table 2) [39]. Therefore, the discrimination into what is considered compliant with the “general strategy of T2T” and not “usual care” is problematic.

Harrold et al. reported findings from a multicenter trial comparing T2T and usual care, which unexpectedly showed treatment adjustments and clinical outcomes were similar across groups (details in Table 2) [25]. This particular study illustrates other confounds of investigating provider adherence; the inclusion criteria of centers having to “agree” to implement T2T, and the provider awareness of the ongoing study itself.

Adherence to T2T also remains a process to which the associated clinical benefits are related to the effectiveness of a treatment regimen in a particular demographic. Unfortunately, the current drugs are not universally effective, nor without adversity, therefore T2T adherence will not always yield satisfactory results. It has previously been emphasized that an intuitive association between T2T protocol compliance and beneficial clinical outcomes does not imply causality and requires consideration of other factors, e.g., a more manageable disease course, medication side effects, and patient characteristics [23,26]. This in turn is what makes investigating and delineating the raw effect of adherence to T2T among providers difficult. 

## 7. Lag Time in Real-World Institution of Practice Guidelines 

Some studies have described that institution and adherence to T2T guidelines occurred shortly following their appearance, attributing favorable control of disease activity and remission rates to this strategy, which was substantiated by an annual overview prior to and shortly after publication [38]. A retrospective chart review study has previously shown that disease activity and functional assessments increased in number from 2010 to 2012, with excellent treatment and management adherence to quality indicators [41]. In a recent systematic review of real-world T2T evidence [13], it has been shown that over time the rates of achieving remission have gradually improved, though this cannot be attributed to T2T alone. It should be noted that alongside T2T, novel drugs and trends in therapy are being introduced, which may also account for the improvement in disease control.

Considering some reports of suboptimal RA management (see Section 2), it may be argued that a delay in the institution of “T2T philosophy” will require a few years to significantly change office-based practice, and therefore a lag time will naturally occur before T2T is prevalent. However, studies from a large United States registry have reported that care in concordance with American College of Rheumatology guidelines is not associated with time since their publication [42]. Other reports examining recommendation adherence based on prescription decisions, prior to and after publication of guidelines for disease activity-driven therapy, revealed that providers may still be more inclined to apply less aggressive therapy, even in patients with uncontrolled RA [43]. Meanwhile, adequate control of disease activity is a principle concern in RA care. 

## 8. Clinical Inertia and Comorbidity

Studies show that patients with active RA may not be managed in consistency with recommendations, which advocate intensification of therapy in uncontrolled disease [16]. This phenomenon has been termed as “clinical inertia”, and is increasingly recognized in rheumatology [42], though it has been reported for a variety of chronic conditions, e.g., hypertension, dyslipidemia, or diabetes [44]. The recently updated T2T guidelines have underscored the importance of individual patient-level outcomes, including comorbidity, when undertaking clinical decisions [2]. The T2T strategy recommends a comprehensive approach to the patient, though reducing the inflammatory burden of RA should be a priority. International studies have shown cardiovascular (CV) comorbidity is the most prevalent in RA [45], while other have indicated that it may also shape a difficult-to-treat patient profile [46]. Meanwhile, observational data indicate that in RA patients screening for CV disease prevention is similar to the general population despite a substantially higher vascular risk [47,48]. It may be that hesitation or lower familiarity with comorbidity may lead rheumatologists to “inertia” in refraining from screening and CV prevention. Although, some reports show that CV risk factor recognition and prevention is high [49]. The phenomenon of clinical inertia requires further study in RA. Particular attention should be given to comorbidities such as interstitial lung disease, which is second to CV disease with regard to mortality, while no optimal treatment has been determined, and awareness may be lower [50]. 

## 9. Communication and Personalized Care

Planning and enacting the strategy for treatment requires a degree of mutual understanding, which is difficult to build when there are discordant views, particularly on disease activity and therapy. Nonadherence to T2T owing to patient preferences and differences in patient–provider assessment of disease impact were outlined in a TRACTION trial report [51], which is consistent with other studies [30,52,53]. Although patient choice is undeniably important, it should not pose a significant barrier to optimal treatment strategies. Communicating the importance of abrogating inflammation, while prioritizing health-related quality of life and patient outcomes is what makes up a personalized approach, which will likely make the patient more amenable to the “stringency” of T2T. 

Current evidence supports that discordance between patients’ and physicians’ evaluation of RA course often occurs, e.g., in assessments of global activity [54]. There are a variety of factors, such as pain, fibromyalgia, and depression that may well contribute, even with a lack of synovitis in ultrasound [55,56]. This side to chronic disease may often be underappreciated by physicians, while patients may be less inclined to follow through with more intensive treatment regimen if they feel their concerns are not addressed. Studies of patient and physician-based surveys have underscored the importance of communication, to which there are varying perceptions from both sides, with patients fears and concerns remaining [57]. Apart from being a key aspect of T2T, the mutual decision-making process may be crucial to consistent and effective T2T implementation. There is evidence to suggest that themes such as patient anxiety over therapy [15], pain and impaired mental function [58,59], psychosociological needs [60], and other nonspecific symptoms, e.g., fatigue [61], are important to individual-oriented management. 

It has been noticed that while disease control has improved over recent years, many of these patient-centered themes have not improved, with poor indicators of quality of life [62]. Addressing the patients’ needs in these “challenging” areas is significant for the physician building a working relationship, which may ultimately improve the adherence to more rigorous treatment, benefiting the patient in the long-term. Patient-recorded outcome measures pose a useful tool to determine the impact of RA on patient-centered outcomes of high importance, e.g., pain or fatigue, which may then on be more easily evaluated, quantified, tracked over time and addressed in the treatment plan [62]. Patient assessments offer time and cost-efficiency, location independence, alongside a friendly mode of following RA course, which may also be more actively engaging [63]. Moreover, they are less prone to bias than the often-lengthy recall period at consultation and may more accurately provide an overview of trends in disease. There is a varying extent of validation for some of these measures, which has been outlined elsewhere [63]. 

## 10. Interventions to Improve Adherence and Future Perspectives

Physicians themselves have to be aware, understand, and agree with T2T in order to remain pro-active in this strategy. Studies have shown some benefit from educational interventions in improving physician adherence to guideline elements in rheumatology, though the benefits are moderate [64,65]. TRACTION trial sub-analyses showed a significant difference in T2T adherence between physicians and participating centers [39]. Rheumatologist experience was also noted as a significant factor influencing adherence, which is confirmed by data from observational studies [16]. Considering variability in practice occurs on an individual level, some practitioners may only need to re-familiarize themselves with the strategy, while others will have to acquire more information on the evidence, benefits, and core principles. A randomized trial has shown that a learning collaborative offers the potential to improve T2T adherence among rheumatologists (details, see Table 2) [40]. Lesuis et al. have also discussed the usefulness of registries with real-time feedback on quality care, which may also provide benchmarks for internal reference in healthcare [16].

The T2T strategy advocates a patient-centered approach that may more easily be achieved by an interdisciplinary team. Rheumatologists could acquire the aid of other medical staff in more time-consuming assessments, at the same time incorporating the input from patient questionnaires, while reaching out for specialist advice in treating comorbidity. Pilot studies of nurse calculations of composite disease measures, i.e., DAS28, indicate that although potentially helpful, there is no substantial evidence to support this intervention alone [66]. However, there are several lessons to be learned from the DREAM cohort, where consensus over recommendations was achieved by all practicing rheumatologists, and strategy of care pre-planned to limit overtime during clinical visits [29]. This indicates that alongside a learning collaborative, centers could benefit from an organizational framework to adopting T2T. 

Over two decades ago, practice patterns in RA care were observed among rheumatologists, with substantial individual differences and a tendency to overuse certain clinical measures [67]. It seems that despite the wide endorsement of T2T reported in multinational surveys [68], individual practice patterns that may be discordant from the recommended approach remain a valid concern. We are inclined to think that changes to optimize practice will be difficult to implement as adherence to structured advice (i.e., T2T protocol) has been shown to decline over time, even in the setting of a controlled experiment. Education on the importance of T2T and patient-oriented care will likely have to be stratified on an individual physician-level, in which actual practice and consistent feedback may enable assimilation through personal experience. Digitalized tools may be appropriate not only for determining adherence to T2T, but also for automatized calculation of composite indices, and their incorporation into office-level, electronic records that may provide real-time input for the physician. Designing interventions to improve guideline implementation into practice, rather than awareness and agreement alone, are important to address in future research. Moreover, clarification of real-world cost-efficiency is another crucial aspect. Healthcare centers could provide an internal registry with quality control, with an appropriate structure of care to facilitate frequent inter-physician collaboration. 

## 11. Conclusions

Real-world data and sub-analyses of trials in RA indicate physician adherence to treat-to-target recommendations or relevant elements of protocol is not universal, differs between particular components, decreases over time, and in some cases may be sub-optimal. Evaluating barriers to T2T and/or guideline adherence in routine care is difficult; remembering that individual preferences, clinical characteristics, and the patient–provider relationship play an important role. Understanding of and justification why T2T is not upheld requires not only on a patient’s or physician’s view, but also a healthcare system perspective. Focusing on the patient throughout management requires adequate communication and mutual understanding, which may be improved through utilizing patient-reported outcome measures. Finally, different modes of education may facilitate an improvement in adherence to T2T among providers. 

## Figures and Tables

**Table 1:** Overview of observational studies with relevance to treat-to-target (T2T) adherence among physicians.

Reference	Design	Characteristics	Population (n)	Follow-Up	Study Aim(s)	Outcome(s)	Key Findings
Vermeer et al., 2012, Arthritis Res Ther [29]	longitudinal, observational multicenter (DREAM cohort)	early RA, DMARD naïve	100	28 m	Medical chart review to assess T2T; systematic monitoring with DAS28 and following treatment advice, evaluating deviations, and reasons for nonadherence	(i) visits when DAS28 was determined (ii) visits when therapy was adjusted accordingly to advice (remission yes/no) (iii) most frequent deviation from medication advice (remission yes/no)	(i) 98% of total visits had DAS28, of these 88% agreed with T2T monitoring (ii) 69% of total visits, with remission 80% followed, w/o 58% (iii) tapered/discontinued when it should be continued (remission), no intensification (non-remission)
Escalas et al., 2012, Ann Rheum Dis [37]	longitudinal, observational multicenter (ESPOIR cohort)	early RA, DMARD naïve	782	24 m	Adherence to 2007 EULAR guidelines and impact on radiographic progression and functional ability	(i) DMARDS in patients at risk of erosive/persistent disease (ii) MTX as first DMARD (iii) remission is target and regular monitoring should drive treatment strategy	For (i–iii), adherence was 78%, 67%, and 52%, respectively, for all three 23%, w/o adherence: OR 1.98; 95% CI 1.08–3.62 for radiographic progression, OR 2.36; 95% CI 1.17–4.67 for increase in HAQ ≥1 at 2 y
Wabe et al., 2015, Int J Rheum Dis [23]	single-center, longitudinal	early RA, DMARD naïve	149	36 m	Extent of compliance with T2T strategy necessary to achieve optimal rates of good response at visits	(i) treatment decisions compliant with T2T protocol (ii) cut-off for good outcome at y 3 (iii) cut-off for worse outcome at y 3	(i) 76% of visits (ii) 81% compliance for DAS28 remission and 71% for LDA (iii) remission and LDA are unlikely if physician compliance <70%
Lesuis et al., 2016, RMD Open [16]	single-center, retrospective	early and longstanding RA	137	12 m	(i) guideline adherence in standard care (ii) variation in adherence on parameter and rheumatologist level (iii) predictors for adherence	7 dichotomous guideline adherence parameters (diagnostics, treatment, follow-up and shared care), guideline adherence on patient and visit level, determinants on patient and provider level	(i–ii) therapy change in active disease —67%, regular outpatient visits with DAS28 assessment—37%, correct interval between outpatient visit—32% (ii) variation among rheumatologist interval of visit—11–43% (iii) several rheumatologist and patient-related factors impact guideline adherence (see reference for details)
Xie et al., 2018, Clin Exp Rheumatol [38]	single-center, retrospective	early and longstanding RA, proportion treatment naïve	704	12 m	Sub-cohort trend analyses for first clinic visit prior to and after 2011, comparison with composite indices	Trends in RA control prior to and after publication of guidelines	Higher proportion of pts with low-disease activity and remission in T2T. Visit frequency in all disease activity stages increased after T2T with higher rate of regular follow-up
Taylor et al., 2018, Patient Prefer Adherence [14]	cross-sectional, multinational, data from Adelphi 2014 Disease Specific Programme	early and longstanding RA	2536	N/A	Implementation of T2T in European centers when comparing pts with RA diagnosis <2 or ≥2 years	Applied strategy (i) no target (ii) target other than remission (iii) target set as remission	Proportion of pts (%) treated with respective strategy in early RA (i) 58%, (ii) 8%, (iii) 34%, and longstanding RA (i) 45%, (ii) 19%, (iii) 36%

(i) Abbreviations: Not applicable (N/A), rheumatoid arthritis (RA), disease activity score using 28-joint count (DAS28), patients (pts), low-disease activity (LDA), treat-to-target (T2T), year (y), month (m), Health Assessment Questionnaire (HAQ), Odds Ratio (OR), confidence interval (CI), European League Against Rheumatism (EULAR), Dutch Rheumatoid Arthritis Monitoring registry (DREAM), Etude et Suivi des Polyarthrites Indifferenciees Recentes (ESPOIR). (ii) Definitions of disease character vary across studies; if studies divided patients by RA course, we adopted a definition of early and longstanding RA where deemed appropriate.

**Table 2:** Overview of studies based on clinical trials with relevance to treat-to-target adherence among physicians.

Reference	Design	Characteristics	Population (n)	Follow-Up	Study Aim (s)	Outcome (s)	Key Findings	Commentary
Harrold et al., 2018, Arthritis Care Res [25]	cluster randomized multicenter controlled trial [NCT01407419]	Pts eligible for treatment “acceleration” as assessed by rheumatologist, no criteria for prior medication use or disease duration, moderate to severe RA (CDAI > 10) in standard care	532	every 3 m (total 12 m)	Feasibility and efficacy of T2T vs. usual care	(i) rate of treatment acceleration conditional on CDAI >10 (ii) LDA; CDAI ≤10 achievement	(i) T2T, 47% vs. UC, 50%; OR [95% CI], 0.92 [0.64–1.34]) (ii) LDA achievement T2T, 57% vs. UC, 55%; OR [95% CI], 1.05 [0.60–1.84]	questionnaire-based, intention-to-treat analysis (ii), T2T physicians received prior training, while UC were aware of study aim, outcome assessed on patient level
Yu et al., 2017 Arthritis Care Res [39]	cluster randomized multicenter TRACTION controlled trial [NCT02260778]	early to longstanding RA in standard care	641	4 m for baseline and 4 m for intervention, total 9 m	Adherence to T2T in practice via screening of medical data	(i) specified disease activity target (ii) disease activity record with composite indices (iii) documented shared decision-making (iv) justified treatment decision	(i–v) 64% of visits with no T2T element, 33% with 1, 2% with 2, 0.3% with all (ii) 25% of visits (iii) 39% of visits (iv) 0.3% of visits	data extraction from electronic database of visits, intra- and inter-rater kappa ≥90, external assessors (not self-report) from study staff, outcome not on clinical level but as process-measure (visit level)
Solomon et al., 2017, Arthritis Rheumatol [40]					Impact of learning collaborative on T2T implementation	(i) change in composite T2T score [primary] (ii) positive change in implementation score (% pts) (iii) full implementation of all T2T items (% pts)	(i) baseline for both arms (11%), after 9 months intervention, 57% vs. control, 25% (ii) 84% of pts in intervention arm vs. 37% in control (iii) in follow-up 26% in intervention arm and 6% in control	randomization at site level, unblinded, sample size calculations may not be optimal, primary outcome was not validated previously, little data for baseline disease activity
Kuusaulo et al., 2015, Scan J Rheumatol [22]	randomized, double-blinded, multicenter NEO-RACo controlled trial [NCT0090808]	early, active RA, DMARDs naïve	99	total 60 m (24 m for adherence)	Physician adherence to treatment protocol (CIS score) and clinical outcomes	(i) NEO-RACo remission (ii)DAS28 (iii) radiological joint changes (iv) cumulative work leave (v) DMARD use in y 2–5	(i) 3 m; lower CIS in pts with remission, 0.77 (0.62) vs. w/o 1.46 (0.74), at 2 y 1.83 (1.26) and 3.29 (2.61), respectively (ii) in 2–5 y, higher adherence associated with lower DAS28 than in other groups (iii) no impact on radiological progression (iv) no impact on work leave (v) good vs. low adherence; fewer DMARDs (and bDMARDs)	retrospective analysis, internal consistency for scoring system 0.58 (0.40–0.76), majority of CIS from lack of i.a. GCs, physicians divided into tertiles by adherence, outcome analysis on patient level
Markusse et al., 2016, Arthritis Care & Res [36]	multicenter, randomized, controlled BeSt trial	early, active RA, DMARDs naïve	508	120 m	Evaluation of adherence to DAS-steered T2T strategy in RA with regard to associated conditions	Questionnaire response and T2T adherence; (i) protocol adherence agreement with DAS (ii) and protocol (iii) and RA suppression (iv)	(i) Average 79% in 10-y (100 to 60% at end) (ii) ~80–90% of pts per visit (iii–iv) satisfied with treatment and RA suppression in 75–90% and 85–90% of visits	treatment protocol designed by participating physicians, potential learning curve, and inclusion of younger rheumatologists more accustomed to “T2T”, questionnaire based, some analysis based on hypothetical conditions

(i) Abbreviations: Usual care (UC), treat-to-target (T2T), odds ratio (OR), confidence interval (CI), month (m), year (y), disease modifying antirheumatic drug (DMARD), biologic (b), glucocorticoids (GCs), rheumatoid arthritis (RA), disease activity score (DAS), Low Disease Activity (LDA), Clinical Disease Activity Index (CDAI), new Finnish RA Combination Therapy (NEO-RACo), Behandel Strategieen (BeSt), treat-to-target in RA: Collaboration to Improve adoption and adherence (TRACTION). (ii) Cumulative Inactivity Scale (CIS) is a measure of adherence (lower score, higher adherence, maximum nonadherence of 15).

## References

[1] Taylor P.C., Balsa Criado A., Mongey A.-B., Avouac J., Marotte H., Mueller R.B., Taylor P.C., Balsa Criado A., Mongey A.-B., Avouac J. (2019). How to get the most from methotrexate (MTX) treatment for your rheumatoid arthritis patient?—MTX in the treat-to-target strategy. J. Clin. Med..

[2] Smolen J.S., Breedveld F.C., Burmester G.R., Bykerk V., Dougados M., Emery P., Kvien T.K., Navarro-Compán M.V., Oliver S., Schoels M. (2016). Treating rheumatoid arthritis to target: 2014 update of the recommendations of an international task force. Ann. Rheum. Dis..

[3] Hughes C.D., Scott D.L., Ibrahim F., TITRATE Programme Investigators (2018). Intensive therapy and remissions in rheumatoid arthritis: A systematic review. BMC Musculoskelet. Disord..

[4] Schipper L.G., van Hulst L.T.C., Grol R., van Riel P.L.C.M., Hulscher M.E.J.L., Fransen J. (2010). Meta-Analysis of tight control strategies in rheumatoid arthritis: Protocolized treatment has additional value with respect to the clinical outcome. Rheumatology.

[5] Schoels M., Knevel R., Aletaha D., Bijlsma J.W.J., Breedveld F.C., Boumpas D.T., Burmester G., Combe B., Cutolo M., Dougados M. (2010). Evidence for treating rheumatoid arthritis to target: Results of a systematic literature search. Ann. Rheum. Dis..

[6] Stoffer M.A., Schoels M.M., Smolen J.S., Aletaha D., Breedveld F.C., Burmester G., Bykerk V., Dougados M., Emery P., Haraoui B. (2016). Evidence for treating rheumatoid arthritis to target: Results of a systematic literature search update. Ann. Rheum. Dis..

[7] Brinkmann G.H., Norvang V., Norli E.S., Grøvle L., Haugen A.J., Lexberg Å.S., Rødevand E., Bakland G., Nygaard H., Krøll F. (2019). Treat to target strategy in early rheumatoid arthritis versus routine care—A comparative clinical practice study. Semin. Arthritis Rheum..

[8] Mueller R., Spaeth M., von Restorff C., Ackermann C., Schulze-Koops H., von Kempis J. (2019). Superiority of a treat-to-target strategy over conventional treatment with fixed csDMARD and corticosteroids: A multi-center randomized controlled trial in RA patients with an inadequate response to conventional synthetic DMARDs, and new therapy with cert. J. Clin. Med..

[9] Köhler B.M., Günther J., Kaudewitz D., Lorenz H.-M., Köhler B.M., Günther J., Kaudewitz D., Lorenz H.-M. (2019). Current therapeutic options in the treatment of rheumatoid arthritis. J. Clin. Med..

[10] Littlejohn G., Roberts L., Arnold M., Bird P., Burnet S., de Jager J., Griffiths H., Nicholls D., Scott J., Zochling J. (2013). A multi-center, observational study shows high proportion of Australian rheumatoid arthritis patients have inadequate disease control. Int. J. Rheum. Dis..

[11] Batko B., Stajszczyk M., Świerkot J., Urbański K., Raciborski F., Jędrzejewski M., Wiland P. (2019). Prevalence and clinical characteristics of rheumatoid arthritis in Poland: A nationwide study. Arch. Med. Sci..

[12] Taylor P.C., Alten R., Gomez-Reino J.J., Caporali R., Bertin P., Sullivan E., Wood R., Piercy J., Vasilescu R., Spurden D. (2018). Clinical characteristics and patient-reported outcomes in patients with inadequately controlled rheumatoid arthritis despite ongoing treatment. RMD Open.

[13] Yu C., Jin S., Wang Y., Jiang N., Wu C., Wang Q., Tian X., Li M., Zeng X. (2019). Remission rate and predictors of remission in patients with rheumatoid arthritis under treat-to-target strategy in real-world studies: A systematic review and meta-analysis. Clin. Rheumatol..

[14] Taylor P.C., Alten R., Gomez Reino J.J., Caporali R., Bertin P., Sullivan E., Wood R., Piercy J., Vasilescu R., Spurden D. (2018). Factors influencing use of biologic therapy and adoption of treat-to-target recommendations in current European rheumatology practice. Patient Prefer. Adherence.

[15] Pappas D.A., Gerber R.A., Litman H.J., Gruben D., Geier J., Hua W.D., Chen C., Li Y., Kremer J.M., Andrews J.S. (2018). Delayed treatment acceleration in patients with rheumatoid arthritis who have inadequate response to initial tumor necrosis factor inhibitors: Data from the corrona registry. Am. Heal. Drug Benefits.

[16] Lesuis N., den Broeder A.A., Hulscher M.E.J.L., van Vollenhoven R.F. (2016). Practice what you preach? An exploratory multilevel study on rheumatoid arthritis guideline adherence by rheumatologists. RMD Open.

[17] Roberts L., Tymms K., de Jager J., Littlejohn G., Griffiths H., Nicholls D., Bird P., Young J., Hill J., Zochling J. (2017). The CEDAR study: A longitudinal study of the clinical effects of conventional DMARDs and biologic DMARDs in Australian rheumatology practice. Int. J. Rheumatol..

[18] Batko B., Korkosz M., Juś A., Wiland P. (2019). Management of rheumatoid arthritis in Poland—Where daily practice might not always meet evidence-based guidelines. Arch. Med. Sci..

[19] Kern D.M., Chang L., Sonawane K., Larmore C.J., Boytsov N.N., Quimbo R.A., Singer J., Hinton J.T., Wu S., Araujo A.B. (2018). Treatment patterns of newly diagnosed rheumatoid arthritis patients from a commercially insured population. Rheumatol. Ther..

[20] Gvozdenović E., Allaart C.F., van der Heijde D., Ferraccioli G., Smolen J.S., Huizinga T.W.J., Landewé R. (2016). When rheumatologists report that they agree with a guideline, does this mean that they practise the guideline in clinical practice? Results of the International Recommendation Implementation Study (IRIS). RMD Open.

[21] Tugnet N., Pearce F., Tosounidou S., Obrenovic K., Erb N., Packham J., Sandhu R. (2013). To what extent is NICE guidance on the management of rheumatoid arthritis in adults being implemented in clinical practice? A regional survey. Clin. Med..

[22] Kuusalo L., Puolakka K., Kautiainen H., Blåfield H., Eklund K., Ilva K., Kaipiainen-Seppänen O., Karjalainen A., Korpela M., Valleala H. (2015). Impact of physicians’ adherence to treat-to-target strategy on outcomes in early rheumatoid arthritis in the NEO-RACo trial. Scand. J. Rheumatol..

[23] Wabe N., Sorich M.J., Wechalekar M.D., Cleland L.G., McWilliams L., Lee A., Spargo L., Metcalf R., Hall C., Proudman S.M. (2017). Determining the acceptable level of physician compliance with a treat-to-target strategy in early rheumatoid arthritis. Int. J. Rheum. Dis..

[24] Hifinger M., Hiligsmann M., Ramiro S., Severens J.L., Fautrel B., Watson V., Boonen A. (2017). Patients’ preferences and economic considerations play an important role in treatment decisions: A discrete choice experiment among rheumatologists. Rheumatology.

[25] Harrold L.R., Reed G.W., John A., Barr C.J., Soe K., Magner R., Saunders K.C., Ruderman E.M., Haselkorn T., Greenberg J.D. (2018). Cluster-Randomized trial of a behavioral intervention to incorporate a treat-to-target approach to care of US patients with rheumatoid arthritis. Arthritis Care Res..

[26] Wabe N., Sorich M.J., Wechalekar M.D., Cleland L.G., McWilliams L., Lee A., Spargo L., Metcalf R.G., Hall C., Proudman S.M. (2015). Characterising deviation from treat-to-target strategies for early rheumatoid arthritis: The first three years. Arthritis Res. Ther..

[27] Curtis J.R., Chen L., Danila M.I., Saag K.G., Parham K.L., Cush J.J. (2018). Routine use of quantitative disease activity measurements among US rheumatologists: Implications for treat-to-target management strategies in rheumatoid arthritis. J. Rheumatol..

[28] Kaneko Y., Koike T., Oda H., Yamamoto K., Miyasaka N., Harigai M., Yamanaka H., Ishiguro N., Tanaka Y., Takeuchi T. (2015). Obstacles to the implementation of the treat-to-target strategy for rheumatoid arthritis in clinical practice in Japan. Mod. Rheumatol..

[29] Vermeer M., Kuper H.H., Bernelot Moens H.J., Hoekstra M., Posthumus M.D., van Riel P.L., van de Laar M.A. (2012). Adherence to a treat-to-target strategy in early rheumatoid arthritis: Results of the DREAM remission induction cohort. Arthritis Res. Ther..

[30] Akdemir G., Markusse I.M., Goekoop-Ruiterman Y.P.M., Steup-Beekman G.M., Grillet B.A.M., Kerstens P.J.S.M., Lems W.F., Huizinga T.W.J., Allaart C.F. (2017). Rheumatologists’ adherence to a disease activity score steered treatment protocol in early arthritis patients is less if the target is remission. Clin. Rheumatol..

[31] de Camargo I.A., Almeida Barros B.C., do Nascimento Silveira M.S., Osorio-de-Castro C.G.S., Guyatt G., Lopes L.C. (2016). Gap between official guidelines and clinical practice for the treatment of rheumatoid arthritis in São Paulo, Brazil. Clin. Ther..

[32] Putrik P., Ramiro S., Kvien T.K., Sokka T., Pavlova M., Uhlig T., Boonen A., Working Group (2014). ‘Equity in access to treatment of rheumatoid arthritis in Europe’. Inequities in access to biologic and synthetic DMARDs across 46 European countries. Ann. Rheum. Dis..

[33] Nikiphorou E., Galloway J., van Riel P., Yazici Y., Haugeberg G., Ostor A., Gogus F., Kauppi M., Sokka T. (2017). The spectrum of early rheumatoid arthritis practice across the globe: Results from a multinational cross sectional survey. Clin. Exp. Rheumatol..

[34] Versteeg G.A., Steunebrink L.M.M., Vonkeman H.E., ten Klooster P.M., van der Bijl A.E., van de Laar M.A.F.J. (2018). Long-term disease and patient-reported outcomes of a continuous treat-to-target approach in patients with early rheumatoid arthritis in daily clinical practice. Clin. Rheumatol..

[35] Hetland M.L., Jensen D.V., Krogh N.S. (2014). Monitoring patients with rheumatoid arthritis in routine care: Experiences from a treat-to-target strategy using the DANBIO registry. Clin. Exp. Rheumatol..

[36] Markusse I.M., Dirven L., Han K.H., Ronday H.K., de Sonnaville P.B.J., Kerstens P.J.S.M., Lems W.F., Huizinga T.W.J., Allaart C.F. (2016). Evaluating adherence to a treat-to-target protocol in recent-onset rheumatoid arthritis: Reasons for compliance and hesitation. Arthritis Care Res..

[37] Escalas C., Dalichampt M., Combe B., Fautrel B., Guillemin F., Durieux P., Dougados M., Ravaud P. (2012). Effect of adherence to European treatment recommendations on early arthritis outcome: Data from the ESPOIR cohort. Ann. Rheum. Dis..

[38] Xie W., Li J., Zhang X., Li G., Hao Y., Zhao J., Wang L., Sun X., Fan Y., Zhang Z. (2018). Trends in the activity of rheumatoid arthritis as the consequence of treat-to-target strategy: Eight-year data from 2009 to 2016. Clin. Exp. Rheumatol..

[39] Yu Z., Lu B., Agosti J., Bitton A., Corrigan C., Fraenkel L., Harrold L.R., Losina E., Katz J.N., Solomon D.H. (2018). Implementation of treat-to-target for rheumatoid arthritis in the US: Analysis of baseline data from a randomized controlled trial. Arthritis Care Res..

[40] Solomon D.H., Losina E., Lu B., Zak A., Corrigan C., Lee S.B., Agosti J., Bitton A., Harrold L.R., Pincus T. (2017). Implementation of treat-to-target in rheumatoid arthritis through a learning collaborative: Results of a randomized controlled trial. Arthritis Rheumatol..

[41] Anderson E., Bajaj P., Raghavan S., Patnaik A., Roppelt H. (2016). Assessment of American College of Rheumatology—Endorsed quality indicators in rheumatoid arthritis patients. JCR J. Clin. Rheumatol..

[42] Harrold L.R., Reed G.W., Kremer J.M., Curtis J.R., Solomon D.H., Hochberg M.C., Kavanaugh A., Saunders K.C., Shan Y., Spruill T.M. (2016). Identifying factors associated with concordance with the American College of Rheumatology rheumatoid arthritis treatment recommendations. Arthritis Res. Ther..

[43] Harrold L.R., Harrington J.T., Curtis J.R., Furst D.E., Bentley M.J., Shan Y., Reed G., Kremer J., Greenberg J.D. (2012). Prescribing practices in a US cohort of rheumatoid arthritis patients before and after publication of the American College of Rheumatology treatment recommendations. Arthritis Rheum..

[44] Phillips L.S., Branch W.T., Cook C.B., Doyle J.P., El-Kebbi I.M., Gallina D.L., Miller C.D., Ziemer D.C., Barnes C.S. (2001). Clinical inertia. Ann. Intern. Med..

[45] Dougados M., Soubrier M., Antunez A., Balint P., Balsa A., Buch M.H., Casado G., Detert J., El-zorkany B., Emery P. (2014). Prevalence of comorbidities in rheumatoid arthritis and evaluation of their monitoring: Results of an international, cross-sectional study (COMORA). Ann. Rheum. Dis..

[46] Batko B., Urbański K., Świerkot J., Wiland P., Raciborski F., Jędrzejewski M., Koziej M., Cześnikiewicz-Guzik M., Guzik T.J., Stajszczyk M. (2019). Comorbidity burden and clinical characteristics of patients with difficult-to-control rheumatoid arthritis. Clin. Rheumatol..

[47] Schmidt T.J., Aviña-Zubieta J.A., Sayre E.C., Abrahamowicz M., Esdaile J.M., Lacaille D. (2018). Cardiovascular disease prevention in rheumatoid arthritis: Compliance with diabetes screening guidelines. J. Rheumatol..

[48] Monk H.L., Muller S., Mallen C.D., Hider S.L. (2013). Cardiovascular screening in rheumatoid arthritis: A cross-sectional primary care database study. BMC Fam. Pract..

[49] Castro L.L., Lanna C.C.D., Rocha M.P., Ribeiro A.L.P., Telles R.W. (2018). Recognition and control of hypertension, diabetes, and dyslipidemia in patients with rheumatoid arthritis. Rheumatol. Int..

[50] Spagnolo P., Lee J.S., Sverzellati N., Rossi G., Cottin V. (2018). The lung in rheumatoid arthritis. Arthritis Rheumatol..

[51] Zak A., Corrigan C., Yu Z., Bitton A., Fraenkel L., Harrold L., Smolen J.S., Solomon D.H. (2018). Barriers to treatment adjustment within a treat to target strategy in rheumatoid arthritis: A secondary analysis of the TRACTION trial. Rheumatology.

[52] Tymms K., Zochling J., Scott J., Bird P., Burnet S., de Jager J., Griffiths H., Nicholls D., Roberts L., Arnold M. (2014). Barriers to optimal disease control for rheumatoid arthritis patients with moderate and high disease activity. Arthritis Care Res..

[53] Otón T., Carmona L., Urruticoechea-Arana A., Calvo-Alén J., Arteaga M.J., Cea-Calvo L. (2019). Discordance between doctor and patient assessments and non-adherence to subcutaneous biological drugs. Rheumatol. Int..

[54] Desthieux C., Hermet A., Granger B., Fautrel B., Gossec L. (2016). Patient-Physician discordance in global assessment in rheumatoid arthritis: A systematic literature review with meta-analysis. Arthritis Care Res..

[55] Turk S.A., Rasch L.A., van Schaardenburg D., Lems W.F., Sanberg M., van Tuyl L.H.D., ter Wee M.M. (2018). Pain, sleep and emotional well-being explain the lack of agreement between physician- and patient-perceived remission in early rheumatoid arthritis. BMC Rheumatol..

[56] Challa D.N., Kvrgic Z., Cheville A.L., Crowson C.S., Bongartz T., Mason T.G., Matteson E.L., Michet C.J., Persellin S.T., Schaffer D.E. (2017). Patient-provider discordance between global assessments of disease activity in rheumatoid arthritis: A comprehensive clinical evaluation. Arthritis Res. Ther..

[57] Gibofsky A., Galloway J., Kekow J., Zerbini C., de la Vega M., Lee G., Lee E.Y., Codreanu C., Koehn C., Steinberg K. (2018). Comparison of patient and physician perspectives in the management of rheumatoid arthritis: Results from global physician- and patient-based surveys. Health Qual. Life Outcomes.

[58] Taylor P.C., Moore A., Vasilescu R., Alvir J., Tarallo M. (2016). A structured literature review of the burden of illness and unmet needs in patients with rheumatoid arthritis: A current perspective. Rheumatol. Int..

[59] Grabovac I., Haider S., Berner C., Lamprecht T., Fenzl K.-H., Erlacher L., Quittan M., Dorner T., Grabovac I., Haider S. (2018). Sleep quality in patients with rheumatoid arthritis and associations with pain, disability, disease duration, and activity. J. Clin. Med..

[60] Leon L., Redondo M., Fernández-Nebro A., Gómez S., Loza E., Montoro M., Garcia-Vicuña R., Galindo M. (2018). Expert recommendations on the psychological needs of patients with rheumatoid arthritis. Rheumatol. Int..

[61] Van Steenbergen H.W., Tsonaka R., Huizinga T.W.J., Boonen A., van der Helm-van Mil A.H.M. (2015). Fatigue in rheumatoid arthritis; a persistent problem: A large longitudinal study. RMD Open.

[62] Fautrel B., Alten R., Kirkham B., de la Torre I., Durand F., Barry J., Holzkaemper T., Fakhouri W., Taylor P.C. (2018). Call for action: How to improve use of patient-reported outcomes to guide clinical decision making in rheumatoid arthritis. Rheumatol. Int..

[63] Hendrikx J., de Jonge M.J., Fransen J., Kievit W., van Riel P.L. (2016). Systematic review of patient-reported outcome measures (PROMs) for assessing disease activity in rheumatoid arthritis. RMD Open.

[64] Pope J., Thorne C., Cividino A., Lucas K. (2012). Effect of rheumatologist education on systematic measurements and treatment decisions in rheumatoid arthritis: The metrix study. J. Rheumatol..

[65] Lesuis N., van Vollenhoven R.F., Akkermans R.P., Verhoef L.M., Hulscher M.E., den Broeder A.A. (2018). Rheumatologists’ guideline adherence in rheumatoid arthritis: A randomised controlled study on electronic decision support, education and feedback. Clin. Exp. Rheumatol..

[66] Van Hulst L.T.C., Creemers M.C.W., Fransen J., Li L.C., Grol R., Hulscher M.E.J.L., van Riel P.L.C.M. (2010). How to improve DAS28 use in daily clinical practice?—A pilot study of a nurse-led intervention. Rheumatology.

[67] Henke C.J., Epstein W.V. (1991). Practice variation in rheumatologists’ encounters with their patients who have rheumatoid arthritis. Med. Care.

[68] Haraoui B., Smolen J.S., Aletaha D., Breedveld F.C., Burmester G., Codreanu C., Da Silva J.P., de Wit M., Dougados M., Durez P. (2011). Treating rheumatoid arthritis to target: Multinational recommendations assessment questionnaire. Ann. Rheum. Dis..

